# A novel gene expression system for *Ralstonia eutropha* based on the T7 promoter

**DOI:** 10.1186/s12866-020-01812-9

**Published:** 2020-05-19

**Authors:** Muzi Hu, Bin Xiong, Zhongkang Li, Li Liu, Siwei Li, Chunzhi Zhang, Xueli Zhang, Changhao Bi

**Affiliations:** 1 School of Biological Engineering, Dalian Polytechnic University, Dalian, 116034 People’s Republic of China; 2 Tianjin Institute of Industrial Biotechnology, Chinese Academy of Sciences, Tianjin, 300308 People’s Republic of China; 3 Key Laboratory of Systems Microbial Biotechnology, Chinese Academy of Sciences, Tianjin, 300308 People’s Republic of China

**Keywords:** *Ralstonia eutropha*, *Cupriavidus necator*, T7 expression system, pBBR1 plasmid, Protein expression system

## Abstract

**Background:**

*Ralstonia eutropha* (syn. *Cupriavidus necator)* is a model microorganism for studying metabolism of polyhydroxyalkanoates (PHAs) and a potential chassis for protein expression due to various advantages. Although current plasmid systems of *R. eutropha* provide a basic platform for gene expression, the performance of the expression-inducing systems is still limited. In addition, the sizes of the cloned genes are limited due to the large sizes of the plasmid backbones.

**Results:**

In this study, an *R. eutropha* T7 expression system was established by integrating a T7 RNA polymerase gene driven by the P_BAD_ promoter into the genome of *R. eutropha*, as well as adding a T7 promoter into a pBBR1-derived plasmid for gene expression. In addition, the essential DNA sequence necessary for pBBR1 plasmid replication was identified, and the redundant parts were deleted reducing the expression plasmid size to 3392 bp, which improved the electroporation efficiency about 4 times. As a result, the highest expression level of RFP was enhanced, and the L-arabinose concentration for expression induction was decreased 20 times.

**Conclusions:**

The *R. eutropha* T7 expression system provides an efficient platform for protein production and synthetic biology applications.

## Background

*Ralstonia eutropha*, also known as *Cupriavidus necator*, is a facultative chemolithoautotrophic bacterium which is able to fix CO_2_ via the Calvin-Benson-Bassham (CBB) cycle [1]. It is a model microorganism for studying the metabolism of polyhydroxyalkanoates (PHAs) [1, 2]. When phosphorus or nitrogen was limited, the weight of poly(3-hydroxybutyrate) (PHB) accumulated in the cells exceeded 80% of the cell dry weight [3], which makes *R. eutropha* a potential industrial strain for PHA production.

Metabolic engineering and synthetic biology were used to engineer *R. eutropha* for the production of biofuels and other valued-added products, such as isobutanol [4], 3-methyl-1-butanol [4], methyl ketones [5], hydrocarbons [6], ethanol [7], isopropanol [8, 9], fatty acids [10] and α-humulene [11]. *R. eutropha* is also a potential chassis for production of industrial enzymes and proteins due to its ability to suppress the formation of inclusion bodies [12], lack of acidic byproducts, and high fermentation cell density [3]. When the enzyme organophosphohydrolase (OPH) was produced in engineered *R. eutropha,* its content reached more than 10 g/L, which was a more than 100-fold increase compared to OPH expression in *E. coli* [12]. This indicated that *R. eutropha* is a potential industrial protein production host.

A robust gene expression toolbox which includes suitable host strains, plasmid vectors and promoters is essential for metabolic engineering and synthetic biology. Plasmids derived from pBBR1, and from plasmids of.

IncP and IncQ can replicate in *R. eutropha* individually or compatibly and were used as its plasmid vectors [6]. Promoters used in *E. coli*, such as P_lac_ and P_tac_ [13], were found to be expressed constitutively in *R. eutropha*, while native promoters from *R. eutropha*, such as P_phaC_ [14], P_phaP_ [14], P_phb_ [14], P_acoE_ [14], P_acoD_ [14] and P_pdhE_ [14], were also evaluated to be functional [13, 14]. In addition, promoters induced by L-arabinose [6], L-rhamnose [15], and anhydrotetracycline [16] were used in *R. eutropha*. However, most of the plasmid vectors and promoters mentioned above are not frequently used. Although broad-host-range plasmid vectors derived from pBBR1 with the L-arabinose induced promoter P_BAD_ are currently the most popular ones, the vectors are very large, which limits the sizes of carried genes and decreases plasmid transformation efficiency.

In this study, a gene expression system based on the T7 promoter was constructed to improve the current gene expression technology for *R. eutropha* and make it a better protein expression system.

## Results

### Construction of a T7 gene expression system for *R. eutropha*

The T7 RNA polymerase gene was cloned from *E. coli* BL21(DE3), modified to be driven by the L-arabinose induced promoter P_BAD_, and the *rfp* gene was cloned under the control of a T7 promoter (taatacgactcactataggg). The resulting plasmid pj5_00019, based on pBBR1, was introduced into either *E. coli* S17–1 [17] or *R. eutropha* C5 (*R. eutropha* H16∆*H16_A0006*∆*H16_A0008–9*) [18]. However, we found that *rfp* was expressed in *E. coli* S17–1, but was not expressed in *R. eutropha* C5 with or without L-arabinose induction. We considered that the failure of expression in *R. eutropha* might due to the multiple copies of the T7 RNA polymerase genes expressed from the multicopy plasmid.

**Fig. 1:**
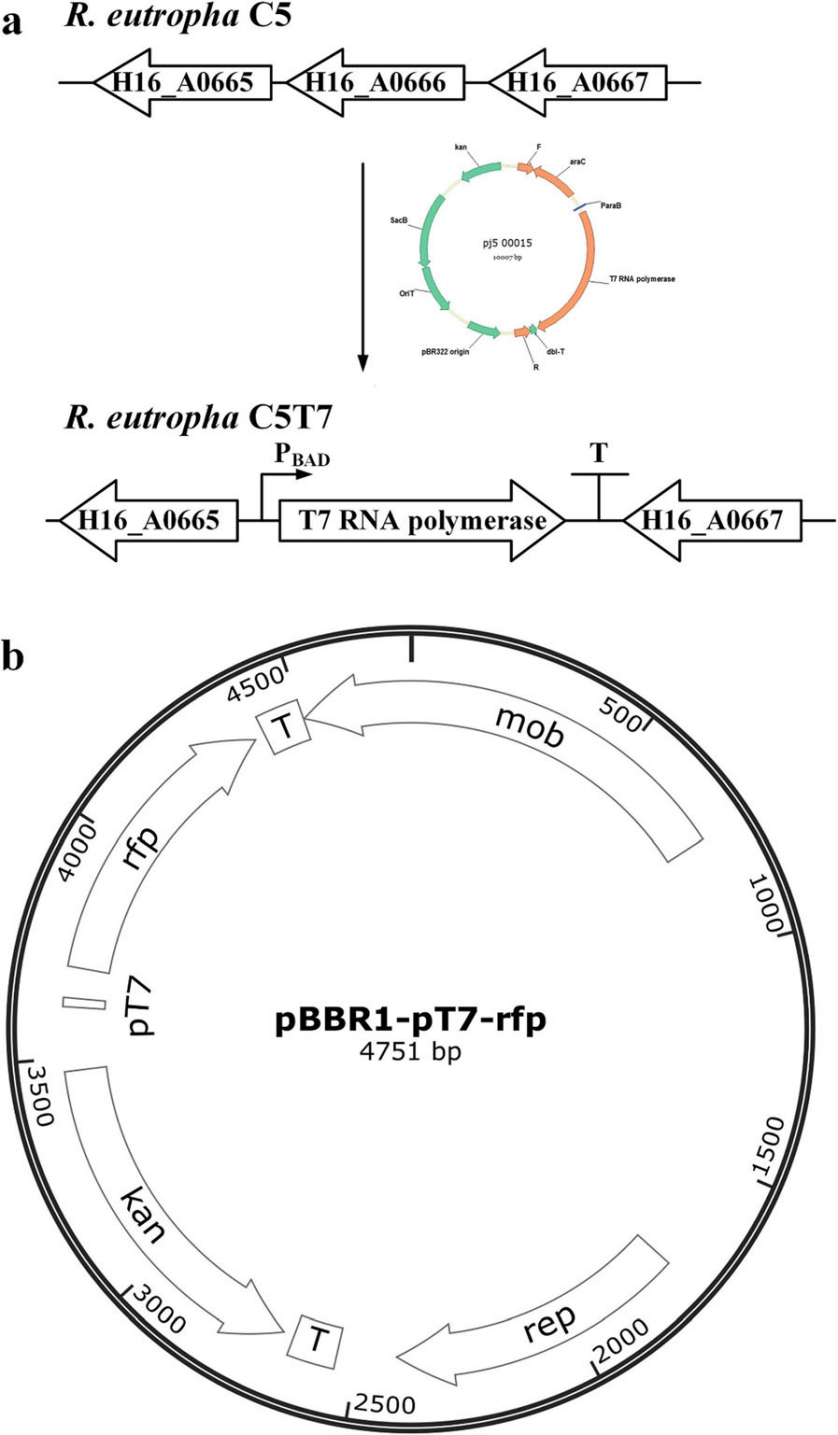
Schematic of the *R. eutropha* gene expression system. The system includes engineered *R. eutropha* C5T7 (**a**) and plasmid vector (**b**). **a** Schematic illustration of the gene arrangment of changed genomic locus of *R. eutropha* C5T7. **b** Profile of gene expression vector pBBR1-PT7-rfp. Bacterial colonies are visually red when *R. eutropha* C5T7(pBBR1-PT7-rfp) is induced by arabinose

To verify the hypothesis, *R. eutropha* strain C5T7 was constructed by integrating the RNA polymerase gene with the P_BAD_ promoter and *araC* into the *H16_A0666* locus on the *R. eutropha* C5 genome. H16_A0666 is annotated as lactate dehydrogenase of *R. eutropha*. Because *R. eutropha* strictly depends on respiratory chain for metabolism, the function of lactate dehydrogenase seems redundant. Therefore, we speculate that the removal of H16_A0666 may not affect its growth, which was verified by the experimental results. The expression plasmid pBBR1-P_T7_-rfp was constructed by cloning the T7-promoter-driven *rfp* into the pBBR1 backbone. After electroporation into *R. eutropha* C5T7, the strain C5T7(pBBR1-P_T7_-rfp) showed visible red color when induced (Fig. 1), which indicated a T7 gene expression system for *R. eutropha* was successfully constructed.

### Minimization of the expression plasmid vector pBBR1-P_T7_-rfp

**Fig. 2:**
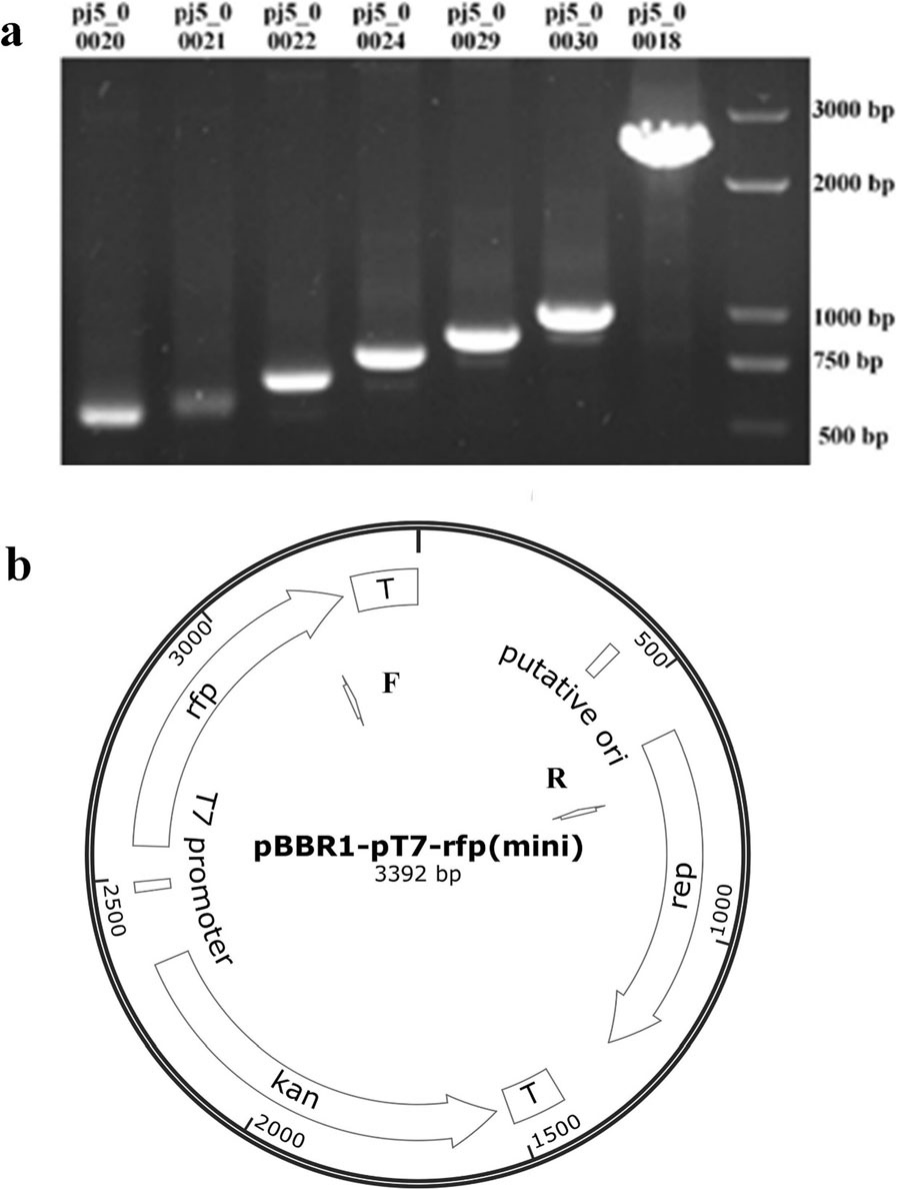
Minimization of plasmid vector pBBR1-P_T7_-rfp(mini). **a** Different plasmids were constructed by deleting various sizes of DNA cassettes from plasmid pj5_00018. PCR were performed using pBBR1_2303F and pBBR1_2303 as primers, which indicated that different sequences were deleted successfully. **b** Profile of the functional minimized plasmid pBBR1-P_T7_-rfp(mini), also named pj5_00030. F: the primer pBBR1_2303F, R: the primer pBBR1_2303R

The pBBR1-P_T7_-rfp plasmid expressed in *R. eutropha* C5T7 had a size of 4751 bp, which limited the size of genes that can be cloned into the vector. Considering the origin of the plasmid, there might be redundant DNA regions that are non-essential for plasmid replication and could be deleted to minimize the plasmid. The DNA cassette containing acttaaaaatcaacaacttaaaaa is AT-rich with two direct repeats, which was assumed to be the replication origin of pBBR1-derived plasmids [19]. Thus, adjacent regions of the putative replication origin were gradually deleted via PCR followed by Golden Gate assembly to reassemble the plasmids. As a result, a series of circular DNA cassettes were constructed, among which only pj5_00030 could be introduced into *E. coli* successfully, which confirmed that only this plasmid contained a functional minimized origin of replication (Fig. 2a). The putative replication origin with about 400 bp of its upstream sequence was conserved in pj5_00030 and was essential for plasmid replication. The minimized plasmid vector pj5_00030 was designated as pBBR1-P_T7_-rfp(mini) (Fig. 2b, Additional file 2).

### Gene expression efficiency of the *R. eutropha* T7 expression system

**Fig. 3:**
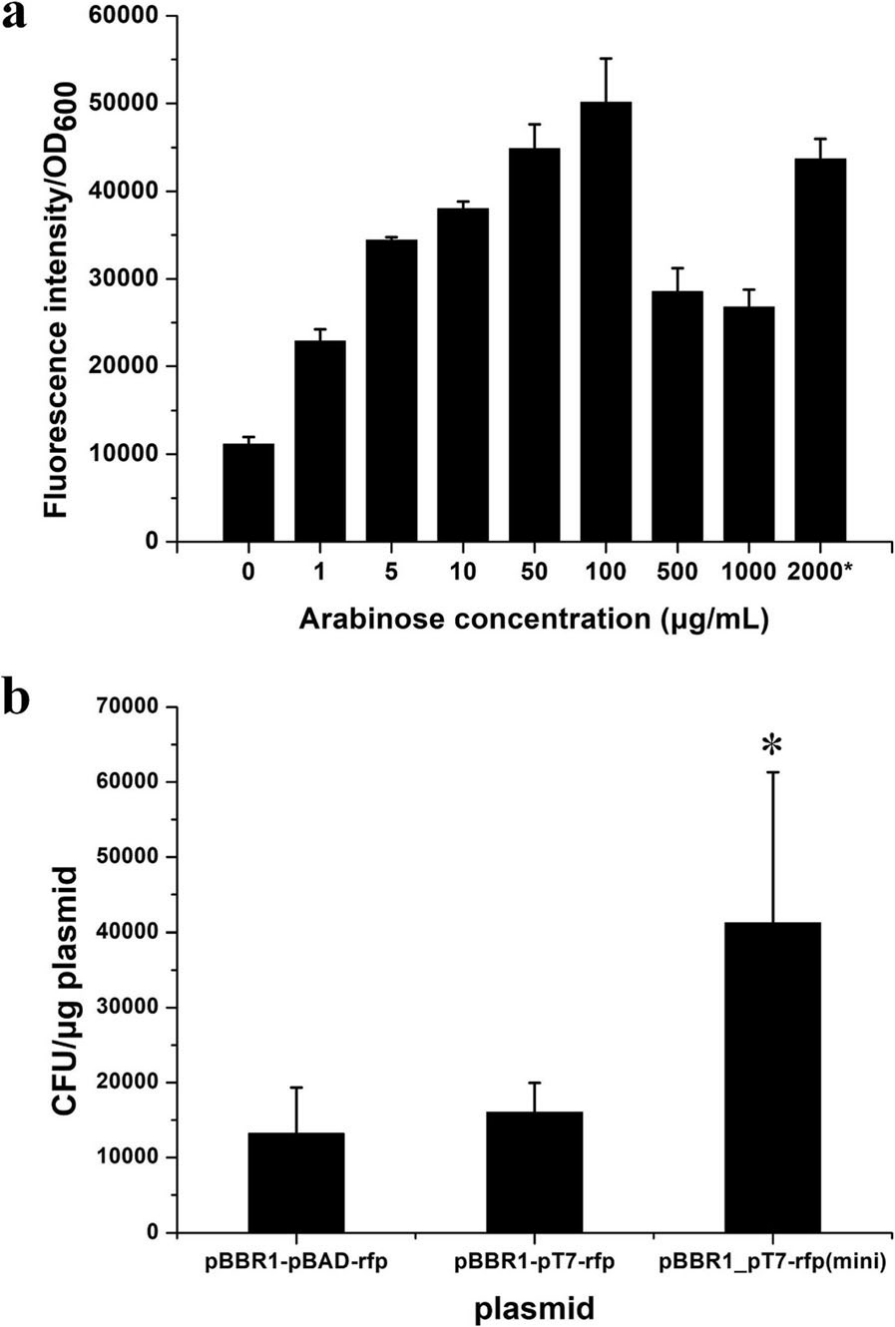
The induced expression strength and electroporation efficiency of pBBR1-P_T7_-rfp(mini). **a** Red fluorescent intensity of *R. eutropha* C5T7(pBBR1-P_T7_-rfp(mini)) with induction of different concentrations of L-arabinose. 2000*: RFP intensity of *R. eutropha* H16 (pBBR1-P_BAD_-rfp) with 2000 μg/mL L-arabinose. **b** electroporation efficiency of pBBR1-P_BAD_-rfp, pBBR1-P_T7_-rfp and pBBR1-P_T7_-rfp(mini) into C5T7 strain. The results represent three biological replicates. The difference was significant (*P* < 0.05)

To determine the performance of the *R. eutropha* T7 expression system with the minimized expression plasmid, pBBR1-P_T7_-rfp(mini) was transferred into *R. eutropha* C5T7 via electroporation for expression analysis. Rfp expression was induced by different concentrations of L-arabinose. The results showed that the expression level of Rfp increased with the increase of L-arabinose concentration in the range from 0 to 0.1 mg/mL L-arabinose. When 0.1 mg/mL L-arabinose was added for induction, the expression level of RFP was highest but decreased when L-arabinose exceeded that threshold (Fig. 3a). Compared with pBBR1-P_BAD_-rfp in *R. eutropha* H16, the highest RFP expression level of *R. eutropha* C5T7(pBBR1-P_T7_-rfp(mini)) was improved. Especially, due to the higher sensitivity of the T7 expression system, the L-arabinose concentration needed for the highest induction level was lowered 20X compared with the traditional P_BAD_ system (Fig. 3a), which can bring significant cost savings in industry.

### Electroporation efficiency of the plasmid

To evaluate the electroporation efficiency of the pBBR1-derived plasmids, pBBR1-P_BAD_-rfp, pBBR1-P_T7_-rfp and pBBR1-P_T7_-rfp(mini) were extracted from *E. coli* S17–1, and then individually electroporated into *R. eutropha* C5T7 competent cells. The results showed that the electroporation efficiency of pBBR1-P_T7_-rfp(mini) was significantly higher than that of pBBR1-P_BAD_-rfp or pBBR1-P_T7_-rfp, probably due to the reduced plasmid size (Fig. 3b). Thus, the minimized vector could provide a better cloning efficiency.

## Discussion

T7 expression system for *R. eutropha* was constructed in this work. This system provides an alternative other than current systems, such as *Escherichia coli* and *Bacillus subtilis*, which also bring some improvements over current systems. The major advantages probably include *R. eutropha*’s ability to suppress the formation of inclusion bodies [12], lack of acidic byproducts, and high fermentation cell density [3]. In addition, since the presence of antibiotics resistance form the plasmid was stable during the all the culturing and experiment process, we considered the expression system was relatively stable.

## Conclusions

In this study, an optimized T7 expression system for *R. eutropha* was constructed for protein expression, synthetic biology manipulation and other applications. The system has a few advantages over previous ones. First, with the high sensitivity of the T7 system, the highest expression level was enhanced, and the L-arabinose concentration for induction was decreased 20 times, offering great cost saving. Secondly, due to the minimized size of the expression vector, the electroporation efficiency was improved 4 times (*p* < 0.05), which also makes the plasmid a better vector for accommodating large DNA cassettes.

## Methods

### Strains and culture conditions

The strains used in this study are listed in Table S1. *E. coli* was cultured at 37 °C in lysogeny broth medium (LB, 10 g/L peptone, 5 g/L yeast extract, 10 g/L NaCl). Streptomycin (100 μg/mL) or kanamycin (50 μg/mL) was added to the medium where appropriate. *R. eutropha* was cultured in LB at 30 °C. Gentamycin (10 μg/mL) or kanamycin (200 μg/mL) was added where appropriate.

### Primers and plasmids

Primers (Table S2) used for the construction of plasmids (Table S3) in this study were designed using j5 Device Editor [20]. The other primers were designed using Clonemanager software. High fidelity DNA polymerase was purchased from Takara (Dalian, China) or New England Biolabs (USA). BsaI restriction endonuclease and T4 ligase were purchased from New England Biolabs (USA) and Thermo-Fisher Scientific (USA), respectively. Plasmids were constructed using Golden Gate [21], Gibson [22] or CPEC [23] methods, and used to transform *E. coli* or *R. eutropha*. Plasmids were extracted using the AxyPrep Plasmid Miniprep Kit (AXYGEN, China) according to the manufacturer’s instructions, with minor modifications.

### Genome integration of *R. eutropha*

Integration of araC and T7 RNA polymerase genes into *R. eutropha* C5 (*R. eutropha* H16∆*H16_A0006*∆*H16_A0008–9*) [18] was performed with the pk18mobsacb suicide plasmid with similar procedure as our previous work [18]. For construction of the integration plasmid pj5_00015, the DNA cassettes of left homologous arm, AraC gene, PBAD promoter, T7 RNA polymerase gene and terminator were assembled into pk18mobsacb plasmid with the Golden gate method. Plasmid pj5_00015 was then transferred into *R. eutropha* C5 strain by bacterial transconjugation. And the strain with kanamycin resistance was screened on agar plates containing 200 μg/ml kanamycin. The strain with genomic integrated pj5_00015 was selected by colony PCR. To eliminate the DNA cassette containing antibiotic marker, the above selected strain was cultured in NaCl free LB liquid media containing 10% sucrose, and then plated agar plate containing the same content to screen for strain with no kanamycin Integration of araC and T7 RNA polymerase genes into *R. eutropha* C5 (*R. eutropha* H16∆*H16_A0006*∆*H16_A0008–9*) [18] was performed with the pk18mobsacb suicide plasmid with similar procedure as our previous work [18]. For construction of the integration plasmid pj5_00015, the DNA cassettes of left homologous arm, AraC gene, PBAD promoter, T7 RNA polymerase gene and terminator were assembled into pk18mobsacb plasmid with the Golden gate method. Plasmid pj5_00015 was then transferred into *R. eutropha* C5 strain by bacterial transconjugation. And the strain with kanamycin resistance was screened on agar plates containing 200 μg/ml kanamycin. The strain with genomic integrated pj5_00015 was selected by colony PCR. To eliminate the DNA cassette containing antibiotic marker, the above selected strain was cultured in NaCl free LB liquid media containing 10% sucrose, and then plated agar plate containing the same content to screen for strain with no kanamycin resistance. The obtained strain was confirmed again with colony PCR and sequencing of the PCR products, which was designated as *R. eutropha* C5T7 strain.

Integration of araC and T7 RNA polymerase genes into *R. eutropha* C5 (*R. eutropha* H16∆*H16_A0006*∆*H16_A0008–9*) [18] was performed with the pk18mobsacb suicide plasmid with similar procedure as our previous work [18]. For construction of the integration plasmid pj5_00015, the DNA cassettes of left homologous arm, AraC gene, PBAD promoter, T7 RNA polymerase gene and terminator were assembled into pk18mobsacb plasmid with the Golden gate method. Plasmid pj5_00015 was then transferred into *R. eutropha* C5 strain by bacterial transconjugation. And the strain with kanamycin resistance was screened on agar plates containing 200 μg/ml kanamycin. The strain with genomic integrated pj5_00015 was selected by colony PCR. To eliminate the DNA cassette containing antibiotic marker, The above selected strain was cultured in NaCl free LB liquid media containing 10% sucrose, and then plated agar plate containing the same content to screen for strain with no kanamycin resistance. The obtained strain was confirmed again with colony PCR and sequencing of the PCR products, which was designated as *R. eutropha* C5T7 strain.

Integration of araC and T7 RNA polymerase genes into *R. eutropha* C5 (*R. eutropha* H16∆*H16_A0006*∆*H16_A0008–9*) [18] was performed with the pk18mobsacb suicide plasmid with similar procedure as our previous work [18]. For construction of the integration plasmid pj5_00015, the DNA cassettes of left homologous arm, AraC gene, PBAD promoter, T7 RNA polymerase gene and terminator were assembled into pk18mobsacb plasmid with the Golden gate method. Plasmid pj5_00015 was then transferred into *R. eutropha* C5 strain by bacterial transconjugation. And the strain with kanamycin resistance was screened on agar plates containing 200 μg/ml kanamycin. The strain with genomic integrated pj5_00015 was selected by colony PCR. To eliminate the DNA cassette containing antibiotic marker, The above selected strain was cultured in NaCl free LB liquid media containing 10% sucrose, and then plated agar plate containing the same content to screen for strain with no kanamycin resistance. The obtained strain was confirmed again with colony PCR and sequencing of the PCR products, which was designated as *R. eutropha* C5T7 strain.

Integration of araC and T7 RNA polymerase genes into *R. eutropha* C5 (*R. eutropha* H16∆*H16_A0006*∆*H16_A0008–9*) [18] was performed with the pk18mobsacb suicide plasmid with similar procedure as our previous work [18]. For construction of the integration plasmid pj5_00015, the DNA cassettes of left homologous arm, AraC gene, PBAD promoter, T7 RNA polymerase gene and terminator were assembled into pk18mobsacb plasmid with the Golden gate method. Plasmid pj5_00015 was then transferred into *R. eutropha* C5 strain by bacterial transconjugation. And the strain with kanamycin resistance was screened on agar plates containing 200 μg/ml kanamycin. The strain with genomic integrated pj5_00015 was selected by colony PCR. To eliminate the DNA cassette containing antibiotic marker, The above selected strain was cultured in NaCl free LB liquid media containing 10% sucrose, and then plated agar plate containing the same content to screen for strain with no kanamycin resistance. The obtained strain was confirmed again with colony PCR and sequencing of the PCR products, which was designated as *R. eutropha* C5T7 strain.

Integration of araC and T7 RNA polymerase genes into *R. eutropha* C5 (*R. eutropha* H16∆*H16_A0006*∆*H16_A0008–9*) [18] was performed with the pk18mobsacb suicide plasmid with similar procedure as our previous work [18]. For construction of the integration plasmid pj5_00015, the DNA cassettes of left homologous arm, AraC gene, PBAD promoter, T7 RNA polymerase gene and terminator were assembled into pk18mobsacb plasmid with the Golden gate method. Plasmid pj5_00015 was then transferred into *R. eutropha* C5 strain by bacterial transconjugation. And the strain with kanamycin resistance was screened on agar plates containing 200 μg/ml kanamycin. The strain with genomic integrated pj5_00015 was selected by colony PCR. To eliminate the DNA cassette containing antibiotic marker, The above selected strain was cultured in NaCl free LB liquid media containing 10% sucrose, and then plated agar plate containing the same content to screen for strain with no kanamycin resistance. The obtained strain was confirmed again with colony PCR and sequencing of the PCR products, which was designated as *R. eutropha* C5T7 strain.

Integration of araC and T7 RNA polymerase genes into *R. eutropha* C5 (*R. eutropha* H16∆*H16_A0006*∆*H16_A0008–9*) [18] was performed with the pk18mobsacb suicide plasmid with similar procedure as our previous work [18]. For construction of the integration plasmid pj5_00015, the DNA cassettes of left homologous arm, AraC gene, PBAD promoter, T7 RNA polymerase gene and terminator were assembled into pk18mobsacb plasmid with the Golden gate method. Plasmid pj5_00015 was then transferred into *R. eutropha* C5 strain by bacterial transconjugation. And the strain with kanamycin resistance was screened on agar plates containing 200 μg/ml kanamycin. The strain with genomic integrated pj5_00015 was selected by colony PCR. To eliminate the DNA cassette containing antibiotic marker, The above selected strain was cultured in NaCl free LB liquid media containing 10% sucrose, and then plated agar plate containing the same content to screen for strain with no kanamycin resistance. The obtained strain was confirmed again with colony PCR and sequencing of the PCR products, which was designated as *R. eutropha* C5T7 strain.

### Assessment of RFP expression levels

Red fluorescence intensity was measured using a microplate reader (Infinite M200 PRO, TECAN) after the strains were induced with L-arabinose for 48 h. The excitation and emission wavelengths were 585 and 620 nm respectively [6]. Optical density at 600 nm (OD_600_) was also measured using the microplate reader. The RFP expression levels were determined as the ratio of the red fluorescence intensity to the OD_600_.

### Identification of the region necessary for plasmid replication

Different primers for Golden Gate were designed using j5 DeviceEditor, and PCR was implemented using the plasmid pBBR1-P_T7_-rfp as template. The products of the PCR were purified using the SanPrep DNA Purification Kit (Sangon Biotech, China), and then cyclized via Golden Gate assembly. As a result, some regions of the pBBR1-P_T7_-rfp were deleted, and the minimized plasmids were used to transform *E. coli*. Transformed colonies would be obtained if the core region for plasmid replication was intact, while transformation would be unsuccessful if the core region were damaged.

### Electroporation of *R. eutropha* and evaluation of transformation efficiency

Competent cells of *R. eutropha* were prepared according to the protocol described in our previous work [18]. Briefly, *R. eutropha* was cultured in 100 mL LB at 30 °C and 200 rpm, then chilled on ice for 5–10 min after the OD_600_ reached 0.8. The cells were collected by centrifugation at 3000×g for 5 min and washed with ice-cold sterile 10% (V/V) glycerol three times. Finally, the cells were suspended in about 0.5 mL 10% glycerol and 0.1 mL portions transferred to sterile 1.5-mL tubes. The prepared *R. eutropha* competent cells were used immediately, or frozen in liquid nitrogen and preserved at − 80 °C for a few weeks.

Electroporation of *R. eutropha* was implemented according to the protocol described in our previous work [18]. Briefly, appropriate plasmids were added to the competent cells and mixed gently. The mixture was transferred into a pre-chilled 2-mm electroporation cuvette (Bio-Rad, USA), and incubated on ice for 5 min, after which electroporation was performed at a voltage of 2.3 kV. Immediately afterwards, 1 mL of LB preincubated at 30 °C was added to the cells. All the cells were transferred to a sterile 1.5 mL tube and incubated at 30 °C for 2 h. Then, a portion of the cells was spread on LB agar plates with appropriate antibiotics, and cultured at 30 °C until colonies were visible. Electroporation efficiencies were calculated as the ratio of colony-forming units (CFU) to the amount of the used plasmid DNA.

## Supplementary information

**Additional file 1.** Table S1, Table S2 and Table S3.

**Additional file 2.** Sequence of pBBR1-P_T7_-rfp (mini).

## Data Availability

We provide supporting and necessary data for publication of the article. All supporting data is present in the article and the supplemental material documents. The sequences of the constructed vectors were in Additional file 2.

## Abbreviations

*R.eutropha*: *Ralstonia eutropha*
PHAs: Polyhydroxyalkanoates
CBB: Calvin-Benson-Bassham
PHB: Poly(3-hydroxybutyrate)
OPH: Organophosphohydrolase
CFU: Colony-forming units
