# Supplementary material for: A novel gene expression system for Ralstonia eutropha based on the T7 promoter
Source: BMC Microbiol. 2020 May 19;20:121. doi: 10.1186/s12866-020-01812-9 (PMC7236105; doi:10.1186/s12866-020-01812-9)
Supplement: Supplementary file 1 — Additional file 1. Table S1, Table S2 and Table S3. [file 12866_2020_1812_MOESM1_ESM.docx]

Table S1 Strains used in this study

| Strains | Description | Source or reference |
| --- | --- | --- |
| *E. coli* S17-1 | Host strain for conjugation, thi pro recA hsdR [RP4-2Tc::Mu-Km::Tn7] Tp^r^ Sm^r^ | R.Simon et al.(1983) |
| *E. coli* BL21(DE3) | F^–^ ompT gal dcm lon hsdS_B_(r_B_^-^ m_B_^-^) λ(DE3 [lacI lacUV5-T7 gene 1 ind1 sam7 nin5]) | Lab stock |
| *R. eutrpha* H16 | Wild type, Gen^r^, ATCC 17699, DSM 428 | Lab stock |
| *R. eutrpha* C5 | *R. eutrpha* H16△*H16_A0006*△*H16_A0008-9* | Andreas Schafer et al.(1994) |
| *R. eutrpha* C5T7 | *R. eutrpha* C5 derived, T7 RNA polymerase gene was integrated to *H16_A0666* | This study |

Table S2 Primers used in this study

| Primers | Sequence |
| --- | --- |
| pj5_00015 construction, for T7 RNA polymerase gene integration | |
| j5_00001_pK18_f | cacaccaggtctcaatactcacattaattgcgttgcg |
| j5_00009_pK18_r | cacaccaggtctcacctgctaaaggaagcggaacacg |
| j5_00010_A0666F_f | cacaccaggtctcacaggccgtggggacggccac |
| j5_00011_A0666F_r | cacaccaggtctcaaacggcggcggtaccatccagc |
| j5_00005_(pj5_00019)_f | cacaccaggtctcacgttatgacaacttgacggctacatc |
| j5_00006_(pj5_00019)_r | cacaccaggtctcacgccaggcatcaaataaaacgaaaggc |
| j5_00007_A0666R_f | cacaccaggtctcaggcgcgctcgatcgctgcttgc |
| j5_00008_A0666R_r | cacaccaggtctcagtatgaagatctccctcaccagcgccc |
| pj5_00019 construction | |
| j5_00028_pBAD_f | cacaccaggtctcagagtcgtattaccaggcatcaaataaaacgaaaggc |
| j5_00019_pBAD_r | cacaccaggtctcatatgtatatctccttcttaaaagatcttttgaattcc |
| j5_00020_(CP001509)_f | cacaccaggtctcacatatgaacacgattaacatcgctaagaacg |
| j5_00021_(CP001509)_r | cacaccaggtctcatagagtcgtattgatttggcgttacgcg |
| j5_00022_(pBAD_rfp)_f | cacaccaggtctcatctataaacgcagaaaggcccaccc |
| j5_00029_(pBBR1-rfp)_r | cacaccaggtctcaactcactatagggagtccacaacggtttccc |
| pBBR1-pT7-rfp construction | |
| j5_00026_pBAD_f | cacaccaggtctcagactcactatagggagtccacaacggtttccc |
| j5_00027_pBAD_r | cacaccaggtctcaagtcgtattagacgtcggaattgccagctggg |
| pj5_00020 construction | |
| j5_00030_(pj5_00018)_f | cacaccaggtctcataaggggggtacgcaacagc |
| j5_00031_(pj5_00018)_r | cacaccaggtctcacttataaacgcagaaaggcccacccg |
| pj5_00021 construction | |
| j5_00032_(pj5_00018)_f | cacaccaggtctcataacttaaaaatcaacaacttaaaaaaggggggtacgc |
| j5_00033_(pj5_00018)_r | cacaccaggtctcagttataaacgcagaaaggcccacccg |
| pj5_00022 construction | |
| j5_00034_(pj5_00018)_f | cacaccaggtctcatatttttgccggagggggagcc |
| j5_00035_(pj5_00018)_r | cacaccaggtctcaaatataaacgcagaaaggcccacccgaagg |
| pj5_00024 construction | |
| j5_00038_(pj5_00018)_f | cacaccaggtctcatagcctcacggcggcgagtgc |
| j5_00039_(pj5_00018)_r | cacaccaggtctcagctataaacgcagaaaggcccaccc |
| pj5_00029 construction | |
| j5_00043_(pj5_00018)_f | cacaccaggtctcataaccgcgcagcggcttggc |
| j5_00033_(pj5_00018)_r | cacaccaggtctcagttataaacgcagaaaggcccacccg |
| pj5_00030 construction | |
| j5_00044_(pj5_00018)_f | cacaccaggtctcatacccatcaatttttttaattttctctggggaaaagcc |
| j5_00045_(pj5_00018)_r | cacaccaggtctcaggtataaacgcagaaaggcccacc |
| pBBR1_2303F | tcccacaacgaagactac |
| pBBR1_2303R | gggtttcctcgcaataag |
| A0666_1116F | tgcggcagggtgcgagtc |
| A0666_1116R | ggctgggctgggtctgagg |
| pK18_1246F | ggaacacggcggcatcagag |
| pK18_1246R | gcagggtcggaacaggagag |

Table S3 Plasmids used in this study

| Plasmids | Description | Source or reference |
| --- | --- | --- |
| pBBR1-pBAD-rfp | pBBR1 derived, *rfp* was drived by pBAD, Kan^r^ | Xiong B et al.(2018) |
| pK18mobsacB | Plasmid for gene deletion or integration, Kan^r^ | R.Simon et al.(1983) |
| pj5_00015 | T7 RNA polymerase gene which was driven by P_BAD_ and [homologous](javascript:;) [arm](javascript:;)s were cloned into pK18mobsacB, used for T7 RNA polymerase gene integration | This study |
| pj5_00019 | pBBR1 derived, T7 RNA polymerase gene was driven by P_BAD_, while *rfp* was driven by T7 promoter, Kan^r^ | This study |
| pBBR1-pT7-rfp | pBBR1 derived, *rfp* was drived by T7 promoter, Kan^r^, also named pj5_00018 | This study |
| pj5_00020 | pBBR1-pT7-rfp derived, 1784 bp which possibly be redundant was deleted | This study |
| pj5_00021 | pBBR1-pT7-rfp derived, 1760 bp which possibly be redundant was deleted | This study |
| pj5_00022 | pBBR1-pT7-rfp derived, 1670 bp which possibly be redundant was deleted | This study |
| pj5_00024 | pBBR1-pT7-rfp derived, 1563 bp which possibly be redundant was deleted | This study |
| pj5_00029 | pBBR1-pT7-rfp derived, 1469 bp which possibly be redundant was deleted | This study |
| pBBR1-pT7-rfp(mini) | pBBR1-pT7-rfp derived, 1360 bp which possibly be redundant was deleted, also named pj5_00030 | This study |
