# Supplementary material for: A novel gene expression system for Ralstonia eutropha based on the T7 promoter
Source: BMC Microbiol. 2020 May 19;20:121. doi: 10.1186/s12866-020-01812-9 (PMC7236105; doi:10.1186/s12866-020-01812-9)
Supplement: Supplementary file 2 — Additional file 2. Sequence of pBBR1-PT7-rfp (mini). [file 12866_2020_1812_MOESM2_ESM.docx]

**Sequence of pBBR1-P_T7_-rfp(mini)**

cccatcaatttttttaattttctctggggaaaagcctccggcctgcggcctgcgcgcttcgcttgccggttggacaccaagtggaaggcgggtcaaggctcgcgcagcgaccgcgcagcggcttggccttgacgcgcctggaacgacccaagcctatgcgagtgggggcagtcgaaggcgaagcccgcccgcctgccccccgagcctcacggcggcgagtgcgggggttccaagggggcagcgccaccttgggcaaggccgaaggccgcgcagtcgatcaacaagccccggaggggccactttttgccggagggggagccgcgccgaaggcgtgggggaaccccgcaggggtgcccttctttgggcaccaaagaactagatatagggcgaaatgcgaaagacttaaaaatcaacaacttaaaaaaggggggtacgcaacagctcattgcggcaccccccgcaatagctcattgcgtaggttaaagaaaatctgtaattgactgccacttttacgcaacgcataattgttgtcgcgctgccgaaaagttgcagctgattgcgcatggtgccgcaaccgtgcggcaccctaccgcatggagataagcatggccacgcagtccagagaaatcggcattcaagccaagaacaagcccggtcactgggtgcaaacggaacgcaaagcgcatgaggcgtgggccgggcttattgcgaggaaacccacggcggcaatgctgctgcatcacctcgtggcgcagatgggccaccagaacgccgtggtggtcagccagaagacactttccaagctcatcggacgttctttgcggacggtccaatacgcagtcaaggacttggtggccgagcgctggatctccgtcgtgaagctcaacggccccggcaccgtgtcggcctacgtggtcaatgaccgcgtggcgtggggccagccccgcgaccagttgcgcctgtcggtgttcagtgccgccgtggtggttgatcacgacgaccaggacgaatcgctgttggggcatggcgacctgcgccgcatcccgaccctgtatccgggcgagcagcaactaccgaccggccccggcgaggagccgcccagccagcccggcattccgggcatggaaccagacctgccagccttgaccgaaacggaggaatgggaacggcgcgggcagcagcgcctgccgatgcccgatgagccgtgttttctggacgatggcgagccgttggagccgccgacacgggtcacgctgccgcgccggtagcacttgggttgcgcagcaacccgtaagtgcgctgttccagactatcggctgtagccgcctcgccgccctataccttgtctgcctccccgcgttgcgtcgcggtgcatggagccgggccacctcgacctgaatggaagcttggattctcaccaataaaaaacgcccggcggcaaccgagcgttctgaacaaatccagatggagttctgaggtcattactggatctatcaacaggagtccaagcgagctctcgaaccccagagtcccgctcagaagaactcgtcaagaaggcgatagaaggcgatgcgctgcgaatcgggagcggcgataccgtaaagcacgaggaagcggtcagcccattcgccgccaagctcttcagcaatatcacgggtagccaacgctatgtcctgatagcggtccgccacacccagccggccacagtcgatgaatccagaaaagcggccattttccaccatgatattcggcaagcaggcatcgccatgggtcacgacgagatcctcgccgtcgggcatgcgcgccttgagcctggcgaacagttcggctggcgcgagcccctgatgctcttcgtccagatcatcctgatcgacaagaccggcttccatccgagtacgtgctcgctcgatgcgatgtttcgcttggtggtcgaatgggcaggtagccggatcaagcgtatgcagccgccgcattgcatcagccatgatggatactttctcggcaggagcaaggtgagatgacaggagatcctgccccggcacttcgcccaatagcagccagtcccttcccgcttcagtgacaacgtcgagcacagctgcgcaaggaacgcccgtcgtggccagccacgatagccgcgctgcctcgtcctgcagttcattcagggcaccggacaggtcggtcttgacaaaaagaaccgggcgcccctgcgctgacagccggaacacggcggcatcagagcagccgattgtctgttgtgcccagtcatagccgaatagcctctccacccaagcggccggagaacctgcgtgcaatccatcttgttcaatcatgcgaaacgatcctcatcctgtctcttgatcagatcatgatcccctgcgccatcagatccttggcggcaagaaagccatccagtttactttgcagggcttcccaaccttaccagagggcgccccagctggcaattccgacgtctaatacgactcactatagggagtccacaacggtttccctctagaaataattttggaattcaaaagatcttttaagaaggagatatacatatggcgagtagcgaagacgttatcaaagagttcatgcgtttcaaagttcgtatggaaggttccgttaacggtcacgagttcgaaatcgaaggtgaaggtgaaggtcgtccgtacgaaggtacccagaccgctaaactgaaagttaccaaaggtggtccgctgccgttcgcttgggacatcctgtccccgcagttccagtacggttccaaagcttacgttaaacacccggctgacatcccggactacctgaaactgtccttcccggaaggtttcaaatgggaacgtgttatgaacttcgaagacggtggtgttgttaccgttacccaggactcctccctgcaagacggtgagttcatctacaaagttaaactgcgtggtaccaacttcccgtccgacggtccggttatgcagaaaaaaaccatgggttgggaagcttccaccgaacgtatgtacccggaagacggtgctctgaaaggtgaaatcaaaatgcgtctgaaactgaaagacggtggtcactacgacgctgaagttaaaaccacctacatggctaaaaaaccggttcagctgccgggtgcttacaaaaccgacatcaaactggacatcacctcccacaacgaagactacaccatcgttgaacagtacgaacgtgctgaaggtcgtcactccaccggtgcttaaggatccaaactcgagtaaggatctccaggcatcaaataaaacgaaaggctcagtcgaaagactgggcctttcgttttatctgttgtttgtcggtgaacgctctctactagagtcacactggctcaccttcgggtgggcctttctgcgtttata
